# Intermuscular coherence during arm movement changes significantly with shoulder abduction and age, but not with limb dominance

**DOI:** 10.3389/fphys.2025.1689084

**Published:** 2026-01-16

**Authors:** Andrew Erwin, Angelo Bartsch-Jimenez, Hesam Azadjou, Grace Niyo, Francisco J. Valero-Cuevas

**Affiliations:** 1 Division of Biokinesiology and Physical Therapy, University of Southern California, Los Angeles, CA, United States; 2 Alfred E. Mann Biomedical Engineering Department, University of Southern California, Los Angeles, CA, United States; 3 Mechanical and Materials Engineering Department, University of Cincinnati, Cincinnati, OH, United States; 4 Escuela de Kinesiología, Facultad de Medicina, Universidad de Valparaíso, Valparaíso, Chile

**Keywords:** alpha-band, beta-band, electromyography, gamma-band, intermuscular coherence, lateralization, movement, upper extremity

## Abstract

**Introduction:**

Intermuscular coherence (IMC) has the potential to become a clinical biomarker to quantify disruptions of shared neural drive to muscles in individuals with upper and lower extremity motor impairments. Here we test whether shoulder abduction, limb dominance and age affect IMC in unimpaired individuals to serve as a baseline for studies with clinical populations.

**Methods:**

Twenty-five unimpaired participants performed an established single-arm reaching task: rotating an ergometer in the horizontal plane while surface electromyography signals were recorded from the biceps, triceps and deltoids arm muscles. We compared IMC within the alpha, beta, and gamma frequency bands across three experimental factors: shoulder posture (neutral vs. abducted), arm (dominant vs. non-dominant), and age (younger {18–42 years. N = 12, 6 female} vs. older {51–74 years. N = 13, 7 female} adults).

**Results:**

We found that there was a significant effect on IMC due to shoulder posture in the alpha-band ( F=22.4 , p=0.0007 ), beta-band ( F=44.6 , $p=5\times 10^{-5}$ ), and gamma-band ( F=57.9 , $p=4\times 10^{-6}$ ). In addition, IMC was lower in the older group and significantly so in the alpha-band ( F=6.6 , p=0.03 ), but not in the beta- (F=4.5,p=0.07) and gamma-bands ( F=0.52 , p=0.42 ). Although the non-dominant arm tended to have higher IMC, no significant differences due to limb dominance were found.

**Discussion:**

We provide what, to our knowledge, is the first overall comparison of patterns of IMC in unimpaired individuals across arms and the adult lifespan to help future studies quantify and interpret disruptions in neuromuscular control. Beyond confirming the expected increase in IMC with shoulder abduction, we critically demonstrate that age significantly affects IMC in the alpha-band associated with propriospinal sensorimotor processes. We speculate this may be a result of spinal reorganization of spinal motor nuclei due to α -motoneurone death with healthy aging. Given the supporting evidence in this study that limb dominance does not significantly affect IMC, common drive to muscles (as quantified by IMC) is likely driven by subcortical processes that predate the neural lateralization of human upper extremity function.

## 1 Introduction

Coherence analysis provides a non-invasive (albeit indirect) method to quantify the strength of shared neural drive to muscles across frequency bands relevant to motor function (Farmer, 1998; Boonstra and Breakspear, 2012; Boonstra, 2013). Shared neural drive to muscles contains a signature frequency component in the action potentials generated by motoneuron unit pools. This signature frequency originates from the oscillatory neural circuits across the neuraxis (e.g., brain, brainstem, spinal cord) that generate the neural drive to muscles for voluntary and involuntary movement. Quantifying task-specific shared neural drive across muscles, sometimes called ‘synergies,’ could enable understanding coordination strategies across brain regions during voluntary movements (Baker et al., 2003; Boonstra, 2013; Farmer, 1998; Farina et al., 2014b; Gross et al., 2002; Grosse et al., 2002; Heckman and Enoka, 2004) and, most importantly, could serve as a biomarker for disruptions in muscle coordination in movement disorders following neurological conditions and stroke.

The use of coherence analysis can help clarify and quantify the concept of ‘synergies’ in health and disease, which have received multiple definitions and passionate interpretations (Alessandro et al., 2013; Bizzi and Cheung, 2013; Cheung et al., 2009; d’Avella and Bizzi, 2005; Singh et al., 2018; Tresch and Jarc, 2009; Kutch and Valero-Cuevas, 2012; Latash, 2009). To bring clarity to the interpretation of synergies, we distinguish *descriptive* from *prescriptive* synergies (Bartsch-Jimenez et al., 2023; Brock and Valero-Cuevas, 2016; Mulla and Keir, 2023; Valero-Cuevas, 2016; Niyo and Valero-Cuevas, 2024). Descriptive synergies are correlations observed when analyzing the activity of multiple muscles without specifying the origin of such correlations, while prescriptive synergies are those that are thought to be the result of actual intended and coordinated common control signals to multiple muscles. Both prescriptive and descriptive synergies can be detected by dimensionality reduction methods (e.g., principal component analysis, non-negative matrix factorization), but the critical difference is that prescriptive synergies can be demonstrated to originate within the nervous system (i.e., have a causal explanation) and are not just correlations in the data (Kutch and Valero-Cuevas, 2012; Brock and Valero-Cuevas, 2016; Valero-Cuevas, 2016). Common drive (as per IMC at specific frequencies) could therefore help quantify the neural origin of prescriptive synergies (Laine et al., 2021). Synergies of neural origin (which we call ‘synergies’ from now on) are conceptually and physiologically distinct from the ubiquitously observed amplitude-based muscle activity synergies found from smoothed (i.e., low-pass filtered) EMG signals to muscles (Kutch and Valero-Cuevas, 2012; Laine et al., 2021; Laine and Valero-Cuevas, 2017).

As a promising approach to quantify synergies of neural origin, intermuscular coherence (IMC) has been widely studied and adopted in studies involving human motor control (Boonstra and Breakspear, 2012; De Marchis et al., 2015; Farina et al., 2014b; Nazarpour et al., 2012; Mohr et al., 2018; Reyes et al., 2017). These motor control studies measured intermuscular coherence during dexterous manipulation (Laine and Valero-Cuevas, 2017), precision grip (Popp et al., 2023), wrist movement (Hu et al., 2018), and whole-arm movement (Laine et al., 2021; Bartsch-Jiménez and Valero-Cuevas, 2025). Across studies, IMC analysis revealed neural synergies that emerge in typical upper extremity function. In dysfunction, IMC has been used to investigate neural synergies in medical conditions such as stroke to evaluate alteration in shared neural drive due to motor overflow (Chen et al., 2018), corticospinal tract integrity (Ko et al., 2023), functional coordination (Liu et al., 2022), and shoulder abduction (Lan et al., 2017).

After hemiparetic stroke, shoulder abduction is known to further compromise the function of the more affected arm by exacerbating ‘pathological flexion synergies’ that tend to shift the resting posture of the arm, wrist, and hand towards the body (Dewald et al., 1995; Lan et al., 2014). It is thought that, in unimpaired individuals, the reticulospinal tract provides descending commands that contribute to ‘gross’ reaching movements using proximal arm segments (as opposed to fine dexterous manipulation of the hand), such as the shoulder and elbow (Brownstone and Chopek, 2018). Thus, these same pathways may be upregulated after stroke in response to damage to the corticospinal tract, manifesting as the pathological flexion synergy (Hammerbeck et al., 2021; McPherson et al., 2018). In a study with stroke survivors, significant coherence was found in the alpha-band (8–16 Hz) between shoulder and wrist/finger muscles when opening or grasping the hand with the shoulder abducted (Lan et al., 2017). Interestingly, the reticulospinal tract has a frequency signature in the alpha-band, as demonstrated by acoustic startle (Grosse and Brown, 2003). Therefore, we propose that IMC in the alpha-band among upper extremity arm muscles could serve as an informative biomarker of altered reticulospinal drive in hemiparetic stroke survivors.

It is for this reason that our group created an arm cycling task that generates stationary data of sufficient duration to allow IMC analysis to quantify shared neural drive during voluntary arm reaching movement with the shoulder either abducted or in a neutral posture (Laine et al., 2021). Motivated by the foundational work of Dewald et al. (1995), we have previously demonstrated an increase in alpha-band IMC with shoulder abduction (Laine et al., 2021; Bartsch-Jiménez and Valero-Cuevas, 2025), but no prior study to our knowledge has set out to describe baseline interactions across limb dominance and age. Since motor impairment in the upper extremity is more pronounced in the contralateral arm innervated by the corticospinal tract of the lesioned brain hemisphere (Dewald et al., 1995; Hammerbeck et al., 2021)—which can be either arm—it is necessary to establish a baseline IMC for both arms. As a critical first step to assess how synchronous neural drive to muscles is disregulated after stroke, here we seek to establish a baseline for whether and to what extent IMC in the alpha-band (8–16 Hz), beta-band (16–30 Hz), and gamma-band (30–50 Hz) changes in unimpaired individuals with (i) shoulder abduction, (ii) limb dominance and (iii) healthy aging.

## 2 Materials and methods

**FIGURE 1:**
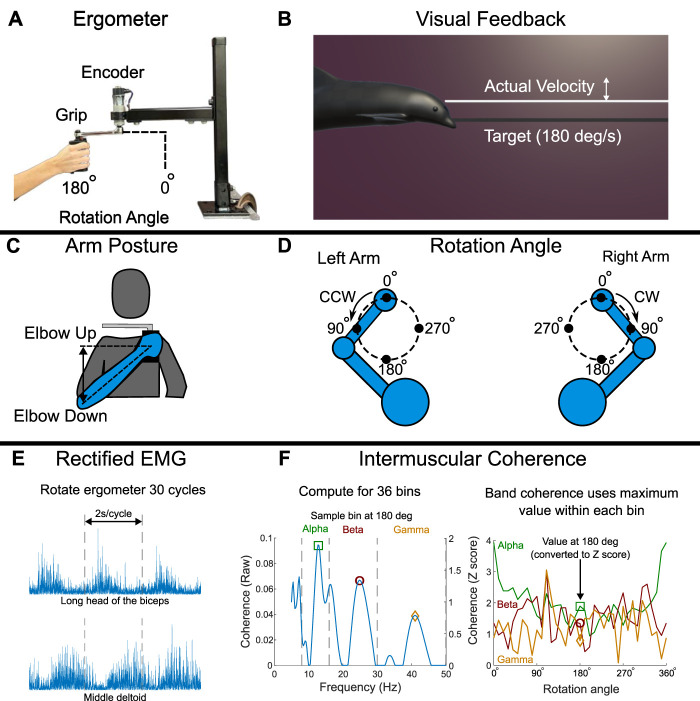
Overview of the single-arm upper extremity motor experiment. **(A)** Participants rotated an ergometer while muscle activity was acquired with sEMG electrodes placed on the biceps, triceps, and deltoid muscles. **(B)** Real-time visual feedback of angular velocity was provided to ensure steady rotation during the task. **(C)** The task was completed in 4 conditions: with the dominant and non-dominant arms and with a neutral (‘Elbow Down’) and abducted (‘Elbow Up’) shoulder posture. **(D)** The rotation angle definitions used in IMC analysis were mirrored across arm conditions so that the same rotation angle represents similar anatomical joint angles for either arm. **(E)** EMG data acquired during the task were high-pass filtered and rectified prior to coherence analysis. **(F)** Intermuscular coherence was computed between two processed EMG signals at each phase of the rotation cycle and then converted to ‘band’ coherence by taking the maximum coherence within each of the alpha-, beta-, and gamma-bands respectively.

We conducted a single-arm cyclical movement experiment to study the extent to which shared neural control of arm movement—as quantified by IMC among arm muscles—is altered by shoulder abduction, limb dominance, and age in unimpaired individuals. The single-arm upper extremity motor task involved rotating a custom ergometer in the horizontal plane for 30 full rotations at a target pace of 2 seconds per rotation cycle. During the task, participants were seated and equipped with surface EMG electrodes placed on the biceps, triceps, and deltoid muscles. Shared neural drive among muscle pairs was quantified through IMC analysis in the alpha-band (8–16 Hz), beta-band (16–30 Hz), and gamma-band (30–50 Hz) frequency ranges. All participants completed the upper extremity task under four experimental conditions to measure the effects of shoulder posture (neutral or abducted) and arm (dominant or non-dominant) on IMC during single-arm movements. Individuals who participated in our study ranged in age across the adult human lifespan (18–74 years), and so we evaluated the effect of age on IMC by comparing across participants sorted into younger (18–42 years) and older (51–74 years) adult subgroups. An overview of the experimental methods is illustrated in Figure 1.

### 2.1 Ethical approval

All procedures were approved by the University of Southern California internal review board (USC IRB: HS-17-00304). Written consent was obtained from each participant prior to starting experimental conditions.

### 2.2 Study participants

We recruited 25 unimpaired individuals (13 females/12 males) with an age range of 18–74 years (mean age ± one standard deviation: 46 ± 17 years) to complete our study. Study participants had the capacity to move their upper extremities and were free of impairments affecting motor control of either arm. Twenty-one participants self-reported as right-hand dominant and four participants self-reported as left-hand dominant. In our analysis, we compare across dominant and non-dominant arms to account for individual differences in limb dominance. Supplementary Table S1 provides additional demographic data for the younger (ages 18–42 years: n=12 ) and older (ages 51–74 years: n=13 ) adult subgroups used in the analysis of age effects. Compared to lifespan definitions used in the literature (Medley, 1980), our age range for younger adults closely reflects early adulthood to middle age, while our age range for older adults is reflective of middle age to late adulthood.

### 2.3 Arm cycling task

To quantify IMC during upper extremity movement in general, participants performed a single-arm cycling task by rotating a custom ergometer. Participants rotated the ergometer by lightly grasping a handle affixed to a mechanical crank that rotated in the horizontal plane (Figure 1A). Throughout the experiment, participants were seated and instructed to maintain a stable trunk and shoulder abduction posture. In addition, muscle activity was recorded from surface EMG sensors placed on the arm completing the task.

The task commenced with the ergometer handle at the furthest point from the participant’s body. To initiate movement in the clockwise direction, a participant needed to flex their elbow and extend their shoulder (in the horizontal plane) in order to move the handle towards their body along the circular ergometer rotation path. Ergometer rotations were made in a clockwise direction for the right arm and in a counterclockwise direction for the left arm. Movements were mirrored across arms so that the anatomical joint angles of each arm were similar. For both clockwise and counterclockwise rotations, the rotation angle (which was measured by an encoder) was defined to increase in the direction of rotation from the starting point (see Figure 1D).

To ensure a smooth and steady rotation, real-time angular velocity was communicated to the user through visual feedback displayed on a computer monitor set in front of the user. The visual feedback consisted of a cartoon dolphin that moved vertically up and down on the display based on actual angular velocity and a stationary line that indicated the target velocity (Figure 1B). The graphical user interface was implemented by a custom Unity3D Engine application. Rotation data was sampled by an Arduino Nano Every circuit board and was streamed to the Unity application using a Python script. A separate data acquisition board collected all signals used in data analysis, which included the surface EMG signals and the ergometer position signal. Both the ergometer and visual display used in this study are newer versions of those used in our group’s prior work (Laine et al., 2021).

Prior to data collection, surface EMG sensors were placed on the arm used to rotate the ergometer. Then, participants were provided with instructions on how to rotate the ergometer and familiarized themselves with the arm cycling task, including the velocity-feedback display. Task familiarization typically lasted 1–3 min. Once the experimental setup and task familiarization were complete, participants performed the single-arm cycling task. For all experimental conditions, participants rotated the ergometer 30 cycles at a rate of 2 s per cycle (which we confirmed after the experiment, although participants did have a tendency to rotate slightly faster as indicated by a grand mean cycle pace of 1.84 s per cycle). This rotation rate resulted in a nominal task completion time of 60 s, which ensured sufficient data for coherence analysis based on our prior work (Laine et al., 2021). After completion of the ergometer task, participants rested for 1–2 min before carrying out the next experimental condition.

### 2.4 Experimental conditions

We implemented a within subjects factorial design to study the main effects of shoulder posture (neutral vs. abducted) and arm (dominant vs. non-dominant) on IMC during arm movement. The two shoulder postures will now be referred to respectively as ‘elbow-down’ for the neutral shoulder posture (which consisted of approximately 45 degrees of shoulder abduction); and ‘elbow up’ for the abducted shoulder posture (which consisted of approximately 90 degrees of shoulder abduction) since the elbow was ‘up’ at the individual’s shoulder height. Both shoulder postures (illustrated in (Figure 1C) were unrestricted and were verbally communicated to the participant during task completion based on visual inspection by the experimenter of participant arm position. For the elbow-up posture, participants were instructed to keep the vertical position of the elbow at their shoulder height. During the experiment, participants were verbally encouraged to keep this position if they departed from it. Limb dominance, as used in data analysis, was self-reported by participants at the beginning of the study.

The four conditions were completed by first selecting either the dominant or non-dominant arm to perform the single-arm cycling task for both shoulder postures; followed by completing the task with the other arm and both shoulder postures. Presentation of conditions were randomized to mitigate possible effects related to order of conditions, such as learning and attention. Specifically, the dominant arm was assigned first to 11 of the 25 participants, and the elbow-up posture was assigned first to 13 of the 25 participants. The entire experiment lasted about 60 min with a standard deviation of 10 min.

### 2.5 Data acquisition

#### 2.5.1 Ergometer rotation angle

We created an instrumented ergometer to standardize arm movement within and across participants. The ergometer consisted of a handle affixed to the end of a 15.2 cm long ‘crank’ (i.e., mechanical link) mounted to a bracket that housed a shaft. The shaft rotated in a ball bearing (i.e., a revolute joint), which resulted in circular motion of the ergometer handle. A hall effect rotary position sensor (RTY360LVNAX, Honeywell Sensing and Productivity Solutions, North Carolina, United States) was mounted to the end of the bracket to incrementally measure the ergometer rotation angle. Between the handle and end of the crank arm, an additional ball bearing was incorporated for ergonomic reasons to accommodate natural wrist rotations throughout the rotation cycle. The ergometer was a passive device without actuators, thereby providing minimal resistance to arm movements. As a result, movements made with the crank were in principle as similar as possible to unconstrained arm movements of the same kind.

#### 2.5.2 EMG recording

Muscle activity was recorded from arm muscles relevant to the task at hand via surface EMG electrodes placed on the long head of the biceps (lbi), lateral head of the triceps (tri), anterior deltoid (adelt), middle deltoid (mdelt), and posterior deltoid (pdelt) muscles. Surface EMG signals were acquired using pre-amplified bipolar sensors with a 20 Hz–460 Hz bandwidth and 1,000x gain (SX230-1,000, Biometrics Ltd., Newport, United Kingdom). The EMG and ergometer position signals were sampled at 1,000 Hz using a DataLINK data acquisition system (DLK 900, Biometrics Ltd., Newport, United Kingdom). The selection of elbow and shoulder muscles in this study is likely only a subset of those relevant to the task at hand. Characterizing the full-dimensionality of arm movement in this upper extremity task would require measuring muscle activity of all arm muscles at each anatomical joint (Cohn et al., 2018), and is beyond the scope of this study.

After all sensors were placed on the surface of the skin, EMG signal quality was visually inspected on real-time plots of EMG data on a computer monitor. Signal quality was verified for each individual muscle during voluntary contraction of each muscle based on their main function. We further verified signal quality by monitoring muscle activity during task familiarization. Electromyography sensors were positioned on the arm according to standard recommendations (Hermens et al., 2000). To mount the sensors, first the arm surface was cleaned with isopropyl alcohol and then sensors were secured to the skin with a double-sided adhesive tape. A ground electrode was placed on a bony protrusion, usually the wrist ulna, on the uninvolved contralateral arm.

As in our prior study (Laine et al., 2021), we also recorded muscle activity from the short head of the biceps and the upper trapezius muscles. Muscle activity from these muscles are not included in this work. From our prior work, the upper trapezius was found to have minimal coherence with other muscles, and the short of the head biceps has similar coherence to the long head of the biceps, but at lower magnitude, thus providing mostly redundant information.

### 2.6 Data analysis

All signal processing and statistical procedures were implemented offline in MATLAB (Mathworks, Natick, MA, United States). IMC values were computed using custom processing functions as the magnitude-squared coherence between two EMG signals (i.e., EMG-EMG coherence). Statistical analyses were implemented using the ‘Statistics and Machine Learning Toolbox’ in MATLAB. The IMC analysis used in this work is similar to that used in our prior work (Laine et al., 2021), which facilitates comparison across studies.

#### 2.6.1 Intermuscular coherence

Measured EMG signals were first digitally processed by a 4th-order high-pass Butterworth filter with a 250 Hz cutoff frequency and zero-phase lag. The high-pass filtered EMG signals were then rectified using the absolute value function (Figure 1E). High-pass filtering and rectification of the EMG signals facilitates calculating coherence by removing motion artifacts and is thought to accentuate motor unit activity, increasing the accurate classification of shared neural drive to muscles (Boonstra and Breakspear, 2012; Farina et al., 2014a; Laine and Valero-Cuevas, 2017). Processed time-domain EMG signals were then converted to a time-frequency representation to facilitate computation of magnitude-squared coherence. Using a time-frequency representation is appropriate given the non-stationary nature (see (White and Boashash, 1990)) of our time-series electromyography data generated during arm movement. Converting time-series data to the time-frequency domain to compute coherence has been used in electroencephalogram studies (Lachaux et al., 1999; Roach and Mathalon, 2008) and in our prior work (Laine et al., 2021).

Time-frequency signals were generated by convolution of time-domain signals with complex Morlet wavelets. We used 7 wavelets that spanned a frequency range of 5 Hz–250 Hz. The practical implementation of the wavelets used a frequency interval of 0.25 Hz in the lower frequencies of interest (5 Hz–50 Hz) and an interval of 5 Hz in the higher frequencies (50 Hz–250 Hz). We found the use of a finer interval for the lower frequency range (used in primary analyses) and the coarser intervals at higher frequencies (used for removing signal bias) to be an appropriate compromise between signal resolution and computation time. At each frequency interval, time-frequency represented signals generated over the 30 rotation cycles were grouped (i.e., concatenated) into 36 phase bins of 10° width. Time-frequency represented data were binned based on the continuous ergometer rotation angle, with the 36 bin centers spaced every 10° from 0° to 350 degrees of the rotation cycle. With a desired cycle rate of 2 s per cycle, this resulted in a nominal 1.67 s of EMG data (about 1,670 samples) per 10-degree phase bin.

Magnitude-squared coherence was computed within each of the 36 phase bins between two muscle activity signals (i.e., IMC), which had been converted to a time-frequency representation. For each phase bin, we computed the maximum IMC value within the alpha (8–16 Hz), beta (16–30 Hz), and gamma (30–50 Hz) frequency bands—which we refer to in short as *alpha-band IMC, beta-band IMC, and gamma-band IMC*, respectively (Figure 1F). To evaluate strength of coherence for each muscle pair throughout the task, most IMC values presented in the Results section have been averaged across the entire rotation cycle within each frequency band. Finally, an aggregate response metric was computed as IMC averaged across the cycle and muscle pairs—which we define as *overall IMC*—to serve as the dependent (response) variable used in group-level statistical analyses.

Prior to computing IMC, magnitude-squared coherence values (which are not normally distributed) were converted to standard Z-scores to facilitate statistical analyses (Baker et al., 2003; Laine and Valero-Cuevas, 2017). The first step was to apply Fisher’s z-transformation by taking the square-root of magnitude-squared coherence (i.e., conversion to a Pearson correlation coefficient) and then applying the inverse hyperbolic tangent function, mathematically described in Equation 1:

$$F_{z}=atanh\left(\sqrt{coh}\right),$$ (1)

where coh is a magnitude-squared coherence (i.e., IMC) value. Assuming that each of the 30 rotations are independent, coherence can then be expressed as a Z score as shown in Equation 2:

$$Z=F_{z}\sqrt{2L}=7.746F_{z}$$ (2)

with L=30 cycles. Any bias introduced when converting coherence values to standard Z scores was removed by subtracting the average coherence between 100 Hz and 250 Hz. At such high frequency intervals, any signal is likely due to measurement noise or irrelevant to our analysis (Laine et al., 2021).

The described signal processing methodology used to compute IMC was repeated for each participant and all experimental conditions. As a result, this procedure generated Z score IMC values for 25 participants, 4 experimental conditions, 36 phase bins, 10 muscle pairs, and 3 frequency bands used to compute the response variable overall IMC used in statistical analyses. A summary of signal processing parameters is provided in Supplementary Table S2.

Contrasts of Z score IMC values across conditions (or groups) measured the effect size (Cohen’s d ) of an experimental factor. When presenting results, we use the following effect size definitions: trivial (<0.2) , small ( ≥0.2 and <0.5 ), medium ( ≥0.5 and <0.8 ), and large (≥0.8) .

#### 2.6.2 Statistical procedures

To evaluate statistical significance of IMC on experimental factors, we ran a within subjects repeated measures ANOVA. The within subjects model evaluated if differences in the response variable of overall IMC were statistically significant due to three experimental factors of shoulder posture (elbow up and elbow down), arm (dominant and non-dominant), and age (younger and older adults). The repeated measures ANOVA was implemented using the function ranova in MATLAB. Statistical significance for all tests was evaluated at the 95% confidence level ( p<α , where α=0.05 ).

The dependent variable (overall IMC) was grouped by experimental condition (within each frequency band) and evaluated to see if the requirements for an ANOVA were met. When the requirements were not met, we applied corrections to the statistical procedure (e.g., Popp et al., 2023). Specifically, we tested whether the dependent variable was normally distributed through the Shapiro-Wilk and Shapiro-Francia normality test; and we tested if the dependent variable had compound symmetry through Mauchly’s test of sphericity. In cases where normality were not satisfied, the data were square-root transformed to increase the degree of normality. When data were found to not satisfy criteria for sphericity, we applied the Greenhouse-Geisser correction to adjust the F -statistic degrees of freedom (within and between subjects) by a multiple ϵ , which tends to increase the p -value reported by the F -statistic.

Post-hoc comparisons were carried out for any statistically significant differences found due to contrasts of main effects (i.e., across posture within the same arm or across arm for the same posture). Specifically, when significant differences were found between conditions, dependent t -tests were used to evaluate significant differences in IMC averaged across the rotation cycle within individual muscle pairs. A Bonferroni correction was applied within each condition to account for multiple comparisons between all 10 muscle pairs by multiplying calculated p values by 10 (i.e., a p -value was significant if 10∗p<α ). To evaluate whether age effects were significant, independent t -tests were computed to compare overall IMC across the two subgroups categorized by age (younger and older adults) within each condition. All statistical procedures were carried out separately for alpha-band, beta-band, and gamma-band IMC.

## 3 Results

### 3.1 IMC across the frequency range

**FIGURE 2:**
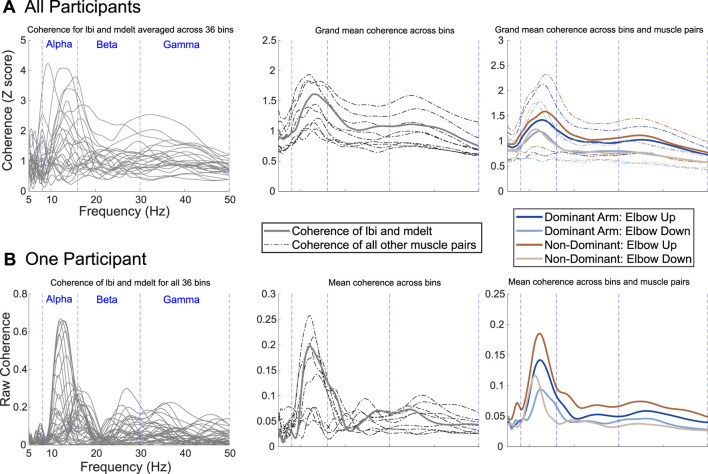
Intermuscular coherence is most pronounced in the alpha-band during arm cycling. Specific and general data are shown to highlight this point across all participants **(A)** and for a sample participant **(B)**. The left column is specific to coherence between the long head of the biceps (lbi) and the middle deltoid (mdelt) muscles. For IMC in the left and center columns, the task was completed with the dominant arm and the elbow up. The plots for the right column show coherence across frequency for all conditions with the grand mean represented by solid lines and one standard deviation by dashed lines.

Grand mean (across all n=25 participants) overall IMC (i.e., coherence averaged across the rotation cycle and muscle pairs) converted to a Z score was greater than 0.5 at each frequency interval within the frequency range of interest (i.e., 8 Hz–50 Hz) for each of the four experimental conditions (Figure 2A, right). This level of grand mean IMC is statistically significant according to Stouffer’s composite Z-score method, which sets 0.33 as the significance threshold at the 95% confidence level (one tail: $1.65/\sqrt{n}$ ). Across the frequency range of interest, the maximum grand mean overall IMC peaked between 11 and 13 Hz suggesting that IMC was strongest in the alpha-band in our arm cycling task (Figure 2A). The peak in grand mean overall IMC within the alpha-band descended into the beta-band (i.e., 16.25 Hz) and reached a valley floor around 20 Hz that then climbed to a smaller secondary peak between 30 and 35 Hz within the gamma-band (Figure 2A). In addition to the IMC results at each frequency interval across participants, IMC for a single participant is shown in Figure 2B within the frequency range of interest.

### 3.2 Statistical significance of overall IMC within each band

Statistical significance on overall IMC (i.e., coherence averaged across the rotation cycle and muscle pairs) due to main effects was evaluated with a repeated measures ANOVA carried out separately for alpha-band, beta-band, and gamma-band IMC (see Supplementary Figure S1), but only after making the following transformations: (i) In each case, overall IMC data were not normally distributed, so we adjusted them by the square root transform; and (ii) Overall IMC data were also found to not satisfy sphericity, and so the Greenhouse-Geisser correction was applied by multiplying F statistic degrees of freedom (which was 1 between groups and 23 within groups) by 0.682 for alpha-band IMC, 0.631 for beta-band IMC and 0.706 for gamma-band IMC. In the reported F statistics that follow, degrees of freedom are omitted for readability. Statistically significant differences in *overall alpha-band IMC* were found due to the main effects of shoulder posture ( F=22.4 , p=0.0007 ) and participant age ( F=6.6 , p=0.03 ). The main effect of limb dominance for alpha-band IMC did not result in significant differences ( F=2.2 , p=0.15 ). Interaction effects that included posture were nearly significant at the 95% confidence level for arm and posture ( F=4.4 , p=0.06 ) as well as for age and posture ( F=3.9 , p=0.08 ) in alpha-band IMC. Differences in *overall beta-band IMC* were significant for shoulder posture ( F=44.6 , $p=5\times 10^{-5}$ ), while differences were not significant for limb dominance (F=1.3,p=0.25) or participant age (F=4.5,p=0.07) . Similarly, differences in *overall gamma-band IMC* were significant for shoulder posture ( F=57.9 , $p=4\times 10^{-6}$ ) but not for limb dominance (F=1.1,p=0.3) or participant age ( F=0.52 , p=0.42 ).

### 3.3 Alpha-band IMC

**FIGURE 3:**
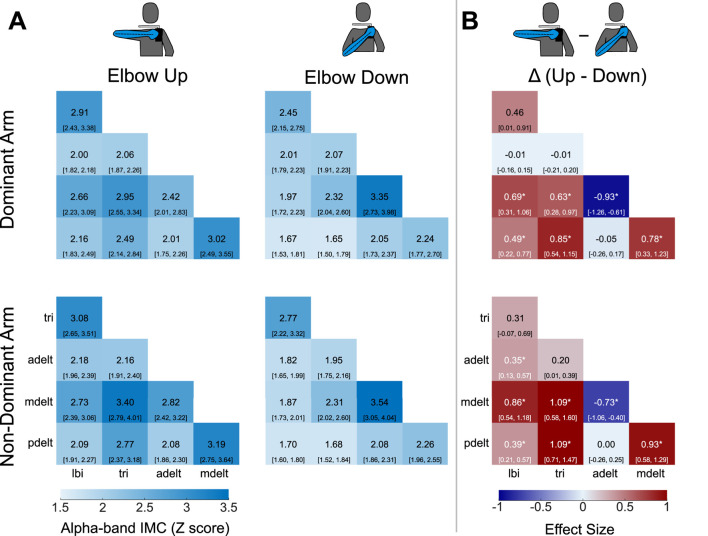
**(A)** Grand mean alpha-band IMC for all muscle pairs and the four experimental conditions. Alpha-band IMC expressed as a Z score was averaged across the rotation cycle (36 bins) within participants, and then the grand mean was computed across all 25 participants. The 95% confidence intervals are included within brackets below the grand mean score in each matrix entry. Arm muscles are abbreviated as the long head of the biceps (lbi), lateral head of the triceps (tri), anterior deltoid (adelt), middle deltoid (mdelt), and posterior deltoid (pdelt). **(B)** Effect size (Cohen’s d ) for the postural contrast of the elbow-up and elbow-down conditions within the same arm (dominant or non-dominant) for all muscle pairs. Significant differences across postures within each arm, denoted by an asterisk, were identified at the 95% confidence level through *post hoc* dependent pair-wise t -tests for each muscle pair using a Bonferroni adjusted threshold for multiple comparisons.

**FIGURE 4:**
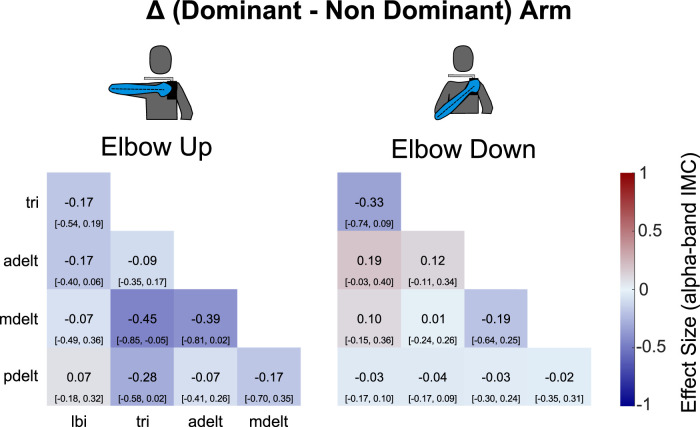
Coherence is not lateralized in the alpha-band when comparing across the dominant and non-dominant arms within the same shoulder posture. Alpha-band IMC for each muscle pair has been averaged across the rotation cycle. A repeated measures ANOVA reported no significant effect of arm on IMC averaged across muscle pairs within each condition. The 95% confidence intervals are depicted in brackets below grand mean scores.

Coherence matrices were created to illustrate alpha-band IMC of all 10 muscle pairs for each experimental condition (Figure 3A). Each entry of a coherence matrix for a given experimental condition displays IMC values expressed as a Z score of the grand mean (across all n=25 participants) and 95% confidence intervals of alpha-band IMC. For these coherence matrices, alpha-band IMC was averaged across the rotation cycle. Effect size contrasts and *post hoc* comparisons due to the main effect of shoulder posture on IMC were computed between all possible muscle pairs (Figure 3B), and contrasts of limb dominance are shown in Figure 4.

Grand mean *overall alpha-band IMC* was 2.65 (standard deviation: 0.69) with the non-dominant arm and the elbow up, 2.47 (0.69) with the dominant arm and the elbow up, 2.20 (0.47) with the non-dominant arm and the elbow down, and 2.18 (0.54) with the dominant arm and the elbow down. Grand mean values reported in the same order in terms of magnitude-squared coherence were: 0.19 (0.06), 0.17 (0.06), 0.14 (0.04), and 0.14 (0.05). Coherence matrices of alpha-band IMC expressed as raw magnitude-squared coherence are provided in Supplementary Figures S2, S3 plots alpha-band IMC across the rotation cycle for several of the significant muscle pairs shown in (Figure 3B). A detailed report of significant differences found between individual muscle-pairs for alpha-band (as well as beta- and gamma-band) IMC are provided in the Supplementary Data Sheet 1.

**FIGURE 5:**
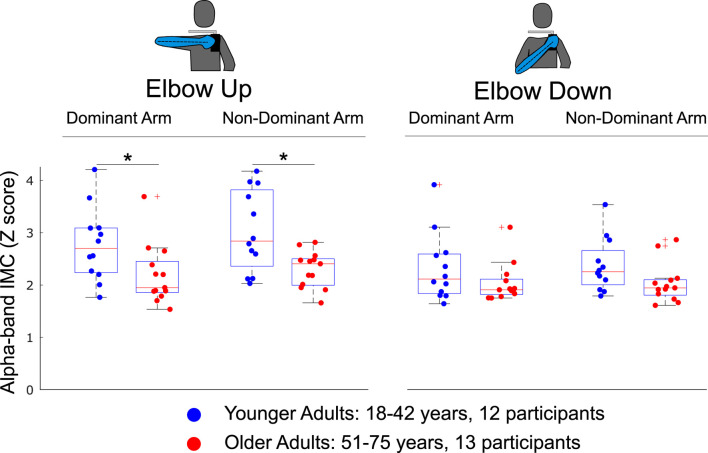
The effect of age on overall alpha-band IMC was evaluated by sorting participants into younger and older adult subgroups. Overall IMC for individual muscle pairs was computed within each condition as the average across the rotation cycle and across all muscle pairs. Alpha-band IMC significantly decreased with age when completing the arm cycling task with the elbow up shoulder posture when using either the dominant or non-dominant arm. Significant differences were not found when completing the task with either arm and the elbow-down. Differences between groups found to be statistically significant through independent t -tests are reported with an asterisk. For the boxplots shown, the red line in the middle of each box is the median, the box edges represent the 25% and 75% quartiles, and the whiskers extend to the extreme values not considered to be outliers (which are indicated by the plus sign symbol). Individual participant values are plotted in front of each box as circles with the younger adult subgroup on the left box and the older adult subgroup on the right box.

The effect of age on *overall alpha-band IMC* was evaluated within each condition across younger and older adult subgroups (see Figure 5). Differences in grand mean *overall alpha-band IMC* were significant between groups when completing the single-arm cycling task with the *elbow-up* posture for both the dominant (p=0.03) and non-dominant (p=0.005) arms. When completing the task with the elbow up, younger adults had a grand mean Z score coherence of 2.8 ( ± 0.7, standard deviation) with the dominant arm and 3.0 with the non-dominant arm (±0.8) ; while older adults had a grand mean of 2.2 (±0.6) with the dominant arm and 2.3 (±0.3) with the non-dominant arm. As a result, comparison across groups with the elbow-up posture yielded medium effect sizes of 0.6 for the dominant arm and 0.7 for the non-dominant arm. Grand mean coherence values tended to be higher for younger adults with the *elbow down*: younger adult grand mean values of 2.3 for the dominant arm and 2.4 for the non-dominant arm compared to grand means from older adults of 2.0 for the dominant arm and 2.0 for the non-dominant arm. Differences between groups with the elbow-down posture, which yielded small 0.3 (dominant arm) to 0.4 (non-dominant arm) effect sizes, were however not statistically significant for either the dominant (p=0.18) or non-dominant (p=0.07) arms. Supplementary Figure S4 plots *overall alpha-band IMC* for each condition with respect to participant age.

### 3.4 Beta-band and gamma-band IMC

**FIGURE 6:**
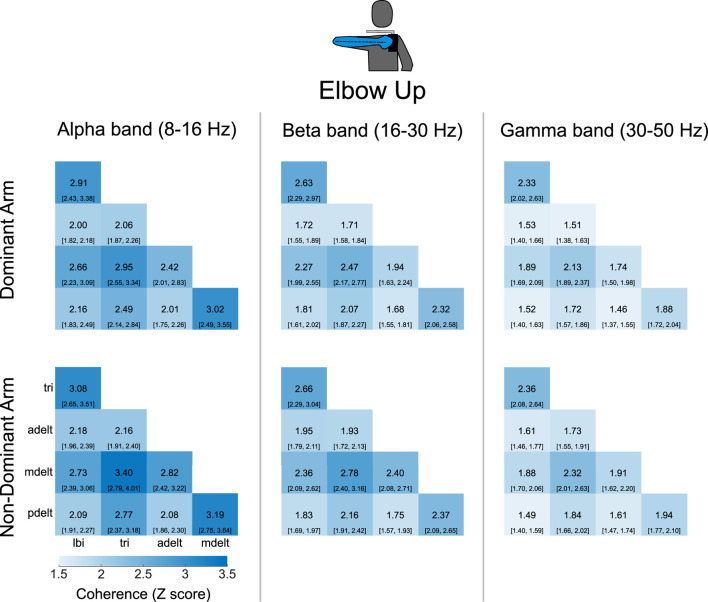
Coherence matrices for alpha-, beta-, and gamma-band IMC were generated for experimental conditions with the elbow-up. Across all muscle pairs, coherence is greatest in the alpha-band, while coherence in the beta-band is higher than the gamma-band. Intermuscular coherence has been averaged across the rotation cycle. Values presented are the grand mean across participants with 95% confidence intervals depicted in brackets.

**FIGURE 7:**
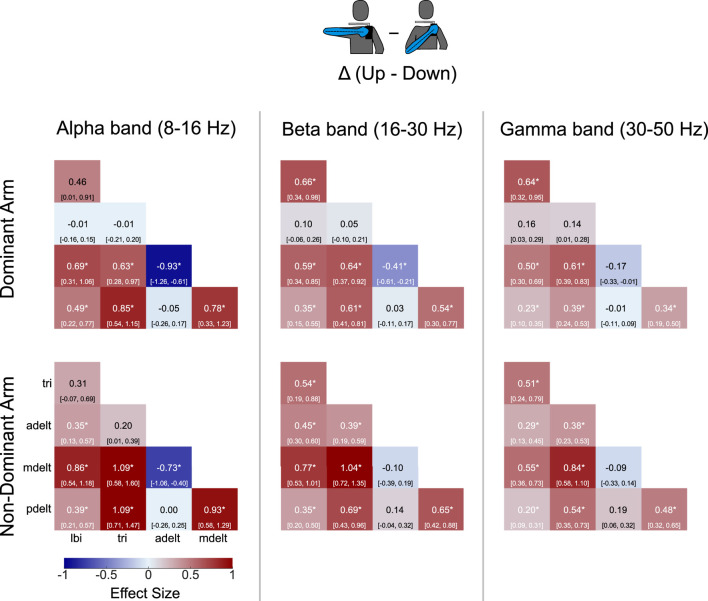
Coherence matrices of grand mean effect size for postural contrasts by and large reveal similar trends across the three frequency bands. IMC for each band has been averaged across the rotation cycle. Grand mean values are presented with 95% confidence intervals depicted in brackets. Significant differences due to posture are identified by an asterisk.

Intermuscular coherence across all muscle pairs within the beta and gamma frequency bands for the elbow-up conditions is also presented in Figure 6 with alpha-band IMC included for reference. The coherence matrices highlight that across all muscle pairs IMC was greatest in the alpha-band, and that beta-band IMC was greater than gamma-band IMC. Differences across the dominant and non-dominant arms had at most small (<0.5) effect sizes within each frequency band. Furthermore, coherence matrices of postural contrasts in all three frequency bands for both arms are presented in Figure 7.

With respect to comparison across age subgroups, for overall beta-band IMC younger adults had small effect sizes with the elbow up of 0.4 (2.27 for younger adults compared to 1.87 for older adults with overall IMC as a Z score) for the dominant arm and 0.3 (2.38 vs. 2.07) for the non-dominant arm. While younger adults had increased overall beta-band IMC with the elbow down for both arms, effect sizes were trivial (0.14 dominant and 0.04 non-dominant). Finally, for overall gamma-band IMC all contrasts yielded trivial effect sizes (magnitude less than 0.16) with older adults having increased coherence (of 0.04) with the elbow down for the non-dominant arms and younger adults having increased coherence for the other three conditions.

## 4 Discussion

Our study evaluated the effects of shoulder posture, limb dominance, and age on IMC within the alpha (8–16 Hz), beta (16–30 Hz), and gamma (30–50 Hz) frequency bands when performing a cyclical analogue to reaching movements in the horizontal plane. This innovative cyclical task allows the collection of EMG signals of sufficient consistency and duration to perform coherence analysis on reaching movements (Laine et al., 2021; Bartsch-Jiménez and Valero-Cuevas, 2025). We defined alpha-band IMC, beta-band IMC, and gamma-band IMC as the maximum value of the magnitude squared coherence within each frequency band of interest evaluated within 10 deg width bins of the rotation cycle. IMC was evaluated for all possible pairings of two functional elbow muscles (the long head of the biceps and lateral head of the triceps) and three shoulder muscles (middle, anterior, and posterior deltoid muscles), which resulted in 36 values of IMC for 10 muscle pairs within 3 frequency bands for 25 participants across 4 conditions. Effects of experimental condition were evaluated on overall IMC (an aggregate metric of IMC averaged across the rotation cycle and muscle pairs for each participant) as well as individual muscle pairs. Finally, the effect of participant age on IMC was evaluated by sorting participants into younger (18–42 years) and older (51–74 years) adult subgroups.

The single-arm cycling task was completed using a 2 × 2 study design with two experimental factors of shoulder posture (elbow up and elbow down) and arm (dominant and non-dominant). In our analysis, age was included as a within subjects experimental factor. Our main findings are that (i) shoulder abduction significantly increased overall IMC—as well as IMC across several functional elbow and shoulder muscles—in the alpha, beta, and gamma frequency bands for both the dominant and non-dominant arms. (ii) Limb dominance did not result in significant differences in overall IMC within any frequency band for either the elbow up or elbow down shoulder postures. And (iii) younger adults had greater overall alpha-band IMC compared to older adults, which were significant with the elbow-up posture for both the dominant and non-dominant arms, but not for the elbow-down posture. However, age effects across groups only approached significance for overall beta-band IMC and were far from significance for overall gamma-band IMC.

It is worth noting that while differences due to limb dominance were not significant, the non-dominant arm tended to have higher IMC than the dominant arm. In addition, interaction effects for overall alpha-band IMC between posture and arm, as well as posture and age, approached significance. These interaction effects may have significant physiological importance related to interconnected neural mechanisms, and may warrant further investigation. Further, our data analysis and results focused on overall IMC, which may mask some effects related to coherence within subsets of the rotation cycle. Evaluating coherence within subsets of the rotation cycle could be valuable to study in future work. The remainder of the Discussion now focuses on results that were significantly different and overall IMC.

### 4.1 Shoulder posture

Across both arms and all frequency bands, IMC significantly increased in 39 of 60 comparisons due to shoulder abduction (17 for the dominant arm, 22 for the non-dominant arm), while 3 comparisons showed significant decreases (all between adelt and mdelt). Interestingly, several muscle pairs had significant increases in all 3 frequency bands with small to large effect sizes: lbi with mdelt, lbi with pdelt, tri with mdelt, tri with pdelt, and mdelt with pdelt. Of note, significant differences involving the anterior deltoid were only due to higher coherence with the middle deltoid with the elbow-down within alpha-band IMC across both arms (medium and large effect sizes), and beta-band IMC with the dominant arm (small effect size). Coherence between the long head of the biceps and short head of the triceps were only significant in beta-band and gamma-band IMC with medium effect size (compared to small effect sizes within alpha-band IMC). In our group’s prior work (Laine et al., 2021), alpha-band IMC was evaluated with the dominant arm in ten young participants with the same task but some minor changes in experimental methods (e.g., visual tracking of a cursor instead of a dolphin, previous version of the ergometer), Laine et al. (2021) reported similar trends in alpha-band IMC with the elbow up (with the dominant arm) with relatively high IMC values found between lbi and tri, mdelt and lbi, pdelt and lbi, tri and mdelt, tri and pdelt, as well as mdelt and pdelt, similar to what we found in this work. For completeness, Supplementary Figure S5 presents smoothed muscle activity (as derived from surface EMG signals) across participants and experimental conditions, which are also similar to that observed in Laine et al. (2021).

### 4.2 Limb dominance

The results from our study suggest that IMC is not lateralized during arm movements as differences were not significant when comparing across the dominant and non-dominant arms for the same shoulder posture (either elbow up or elbow down) within the alpha, beta, or gamma frequency bands. A hallmark trait of behavioral lateralization in humans is limb dominance (Sainburg, 2014), the manifestation of which is often attributed to higher levels of the central nervous system (Haaland et al., 2004). The absence of differences in shared neural drive to arm muscles between dominant and non-dominant arms strongly suggests that ‘lateralization’ or ‘limb dominance’ does not apply to shared control of the reaching movements studied here. That is, their shared drive is likely originating below the cortical mediators of limb dominance (such as the lateral frontal pole prefrontal cortex (Neubert et al., 2014), inferior parietal lobe (Goldenberg and Spatt, 2009) or monosynaptic corticomotoneuronal pathways (Sobinov and Bensmaia, 2021; Lemon, 2008)), as understood to have evolved in humans for dexterous function and tool use (Sainburg, 2014).

### 4.3 Age effects

Beyond confirming the expected increase in IMC with shoulder abduction (most recently reported by Bartsch-Jiménez and Valero-Cuevas (2025) in this same task), we critically demonstrate that age significantly affects IMC in the alpha-band. Specifically, alpha-band IMC significantly decreased in older adults compared to younger adults. With reference to the literature, a study with 92 unimpaired participants found no significant changes of IMC in the beta-band (15–30 Hz) during a pinch-grip task (Jaiser et al., 2016). As a result, the age effect found here might be unique to the alpha-band during voluntary movements. Alpha-band coherence decreasing with age may suggest that neurophysiological effects, such as degeneration of motoneuron synapses (Castro et al., 2023) or loss of proprioception (Ribeiro and Oliveira, 2007), might be related to the functional IMC observed in our task as the alpha-band is associated with propriospinal sensorimotor processes.

We speculate that the decrease in alpha-band IMC found in our study is perhaps a result of spinal reorganization of motor nuclei due to α -motoneurone death with healthy aging (Cruz-Jentoft and Sayer, 2019; Hepple and Rice, 2016; Larsson et al., 2019). One potential mechanism is that α -motoneurone death (usually Type II) is known to produce the adoption of ‘orphaned’ muscle fibers by Type I motoneurones. The reinnervation of muscle fibers by a different type of motoneuron results in fiber type conversion and fiber type grouping (DiStefano, 1993). This would in turn disrupt propriospinal sensorimotor processing by increasing effective innervation numbers in the remaining α -motoneurones, which also no longer receive the appropriate proprioceptive spindle input from the orphaned intrafusal fibers. This would at the very least disorganize the propriospinal mechanisms associated with alpha-band IMC, and potentially reduce its strength. However, other mechanism may be at work. This justifies and motivates further investigations into the mechanisms that explain the change in alpha-band IMC we report, which suggest disruption of propriospinal sensorimotor circuitry in healthy aging.

### 4.4 Study limitations

Like most human subjects experiments, our study had limitations. Although we found many results to be significant, our study could have benefited from recruiting additional participants, in particular for the analysis on limb dominance and participant age. While differences in IMC across limb dominance were not found to be significant, IMC tended to be stronger in the non-dominant arm, and so significant differences might be observed with additional participants. For the age-group analysis, increasing the distribution of participants across the adult lifespan would have been beneficial. In addition, one younger participant was identified as an outlier and excluded from the analysis as their average raw alpha-band coherence across the rotation cycle exceeded 0.8 (Z score of 12) for several muscle pairs. Future work might consider reporting coherence after each task to further investigate such data collection that may have been due to unexpected crosstalk not seen in all other 25 participants included in this paper. With respect to the experimental methods, one limitation is that the shoulder abduction angle was not measured during the experiment. As a result, some of the observed differences may be due to unaccounted for variations in shoulder abduction. Future experiments could use motion capture to measure joint angles to quantify the shoulder abduction angle throughout the experiment, and possibly to present the information in real time to participants. Our study also did not rely on physical constraints (like a wrist splint or trunk strap), which could have introduced unmeasured variability. We found these limitations acceptable and natural, as participants were able to perform the task in a comfortable way that might ease translation to clinical settings.

### 4.5 Future work

Quantifying the strength of shared neural drive to arm muscles in unimpaired individuals during reaching movements is essential to understand the type, strength and source of pathological synchrony of neural drive to groups of muscles following stroke. The impaired movement of the arm contralateral to the brain lesion in hemiparetic stroke—which is exacerbated by shoulder abduction (Dewald et al., 1995)—is thought to come from dysregulation of brainstem-mediated neural drive to groups of muscles (Gross et al., 2002). In unimpaired individuals, shoulder abduction leads to increased coherence across several functional muscles. After stroke, movement of the more affected arm may be exacerbated by shoulder abduction, which might result in a lack of functional coherent drive necessary to smoothly move the arm across the workspace. Our findings underscore the importance of phylogenetically older brainstem mechanisms of limb control associated with alpha-band coherence that predate the emergence of lateralization of arm and hand function in humans (Sainburg, 2014), but which are presumed to influence the clinical presentation of stroke (Krakauer and Carmichael, 2022; Dewald et al., 1995).

The absence of significant differences in IMC between dominant and non-dominant arms in unimpaired adults found in this study serves as an important reference when evaluating (potential) changes in IMC between the paretic and non-paretic arms in hemiplegia. Our results suggest that the shared neural drive we recorded in both arms (particularly in the alpha-band) *likely does not* emanate from the higher levels of the central nervous system presumed to underlie the lateralization of arm and hand function, a hallmark behavioral trait in humans. As a result, any unbalance in alpha-band IMC patterns observed across arms in clinical populations completing our experimental task could predominantly, though not exclusively, be attributed to disruptions of subcortical structures, such as the reticulospinal tract or propriospinal processes, caused by hemiparetic stroke (and not limb dominance). Lastly, the decrease in IMC found in this study with healthy aging is an important consideration when evaluating IMC—of either arm—in stroke survivors. We conclude that IMC increases with shoulder abduction in unimpaired individuals for both the dominant and non-dominant arms across the alpha, beta, and gamma frequency bands. This natural feature of muscle coordination in typical function may provide a pathway for exacerbation after hemiparetic stroke in which shoulder abduction alters and exacerbates typical co-activation patterns quantified in this study.

## Data Availability

The raw data supporting the conclusions of this article will be made available by the authors, without undue reservation.
