# Supplementary material for: Intermuscular coherence during arm movement changes significantly with shoulder abduction and age, but not with limb dominance
Source: Front Physiol. 2026 Jan 16;16:1689084. doi: 10.3389/fphys.2025.1689084 (PMC12855101; doi:10.3389/fphys.2025.1689084)
Supplement: Supplementary file 1 [file DataSheet1.pdf]

Supplementary Material

1 SUPPLEMENTARY TABLES AND FIGURES

1.1 Tables

Table S1. Participant demographics (F: Female, M: Male, R: Right, L: Left)

Demographic	Younger Adults	Older Adults	All Participants
Mean Age ± Std (years)	30 ± 7	60 ± 8	46 ± 17
Age Range (years)	18 to 42	51 to 74	18 to 74
Handedness	12 R | 0 L	9 R | 4 L	21 R | 4 L
Sex	6 F | 6 M	7 F | 6 M	13 F | 12 M

Table S2. Coherence signal processing parameters

Process	Parameter	Value	Units
Acquisition	Sample Rate	1,000	Hz
Band	Alpha	8 to 16	Hz
Band	Beta	16.25 to 29.75	Hz
Band	Gamma	30 to 50	Hz
Filter	Type	Butterworth	
Filter	Cutoff Frequency	250	Hz
Filter	Filter Order	4	
Coherence	Number of Morlet Wavelets	7	
Coherence	Frequency Range (low)	5 to 50	Hz
Coherence	Frequency Interval (low)	0.25	Hz
Coherence	Frequency Range (high)	50 to 250	Hz
Coherence	Frequency Interval (high)	5	Hz
Statistics	Fisher's Z to Z-Score	7.745	

1.2 Figures

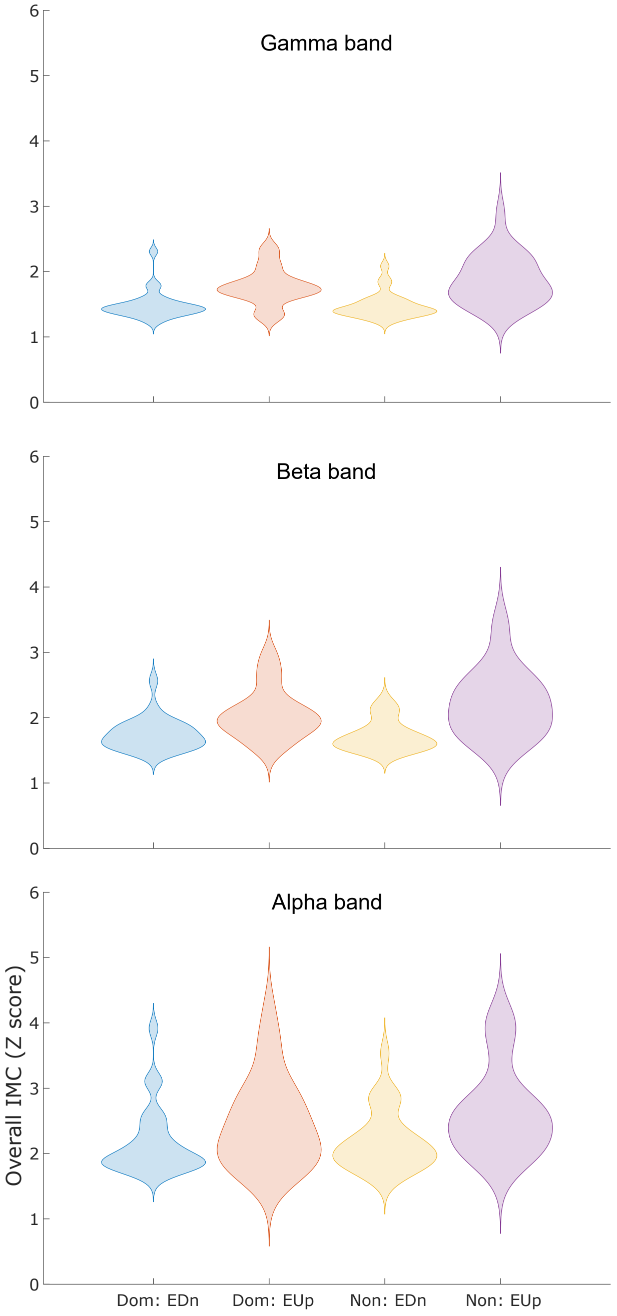

Figure S1. Violin plot for each condition and frequency band of raw intermuscular coherence (i.e., magnitude-squared coherence) averaged across the rotation cycle and muscle pairs (defined here as overall IMC). Conditions are abbreviated in the figure as: Dom: EDn (Dominant Arm, Elbow Down), Dom: EUp (Dominant Arm, Elbow Up), Non: EDn (Non-Dominant Arm, Elbow Down), and Non: EUp (Non-Dominant Arm, Elbow Up). The reader may find the following conversions between raw IMC and Z score values (in parentheses) helpful: 0.01 (0.78), 0.05 (1.76), 0.1 (2.54), 0.2 (3.73), 0.3 (4.76), and 0.4 (5.77).

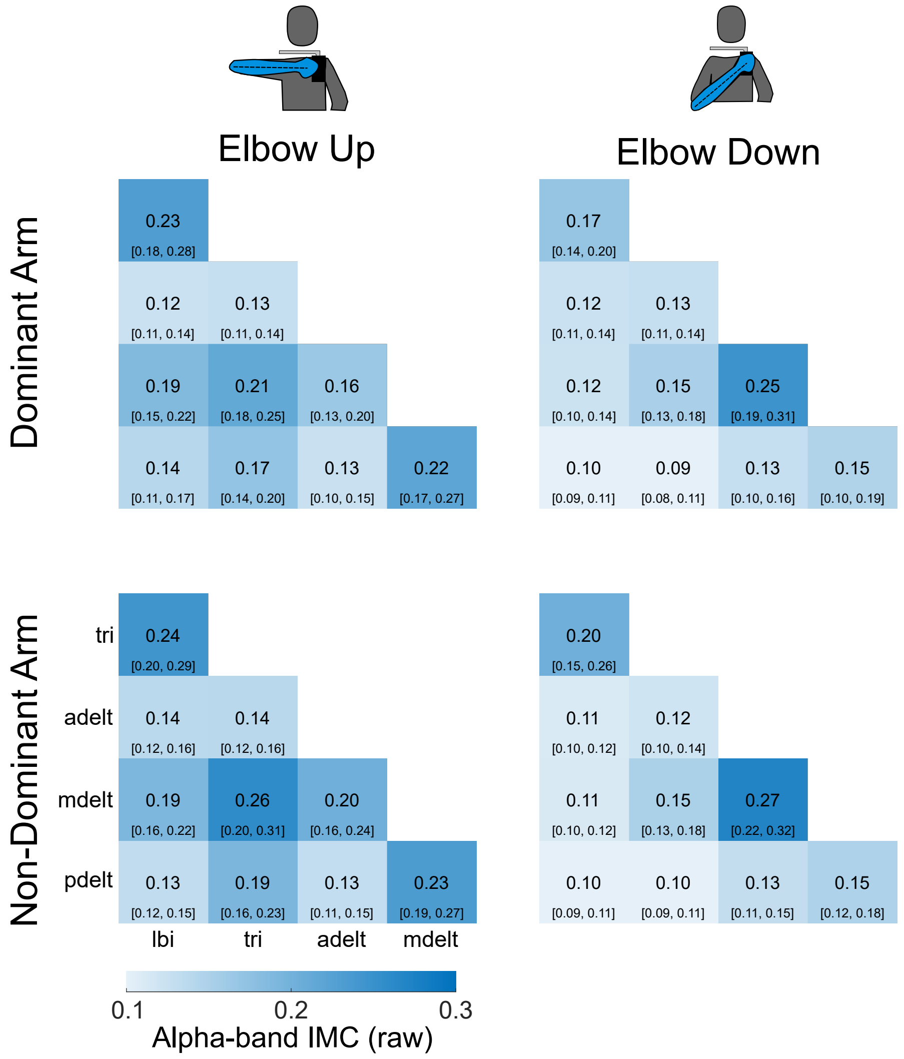

Figure S2. Alpha-band intermuscular coherence for both shoulder postures and arms. Each matrix element represents grand mean raw magnitude-squared alpha-band coherence averaged across the 36, 10 degree-width bins of the rotation cycle.

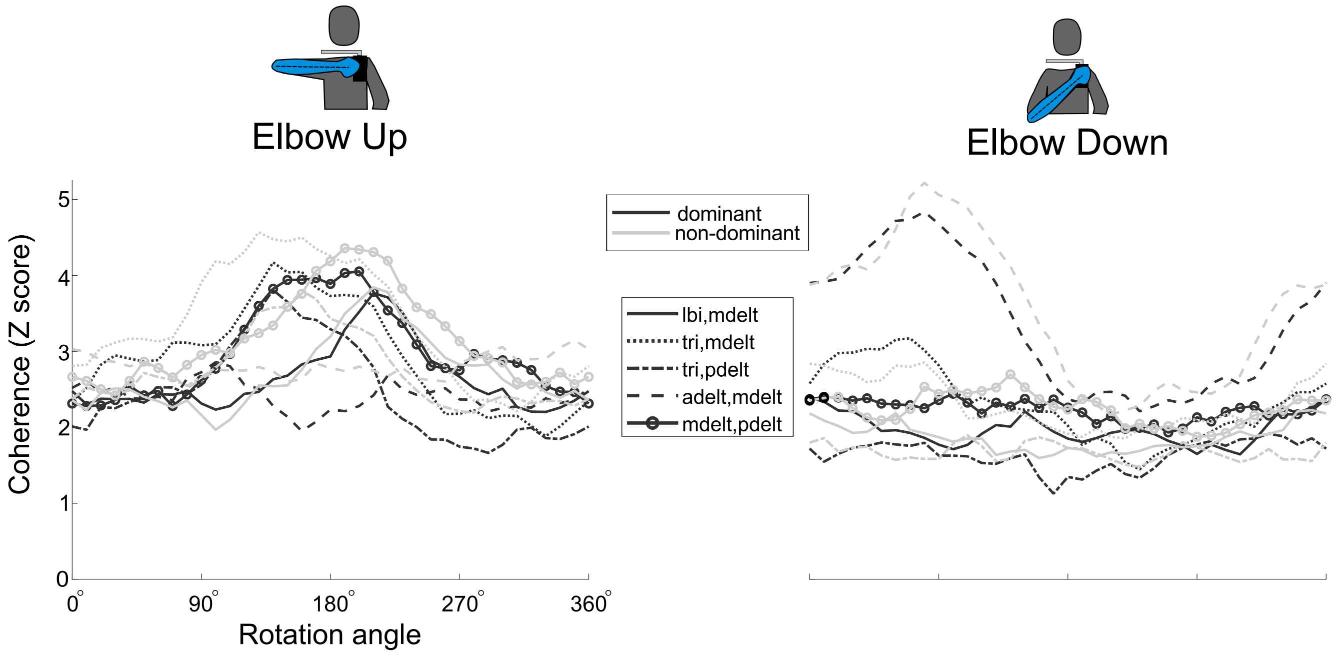

Figure S3. Grand mean coherence across the rotation cycle for muscle pairs that elicited at least a medium effect size (> 0.5) and statistical significance across postures. Coherence varies throughout the rotation cycle with similar trends for the same posture across arms. In the elbow-up conditions, four of the five muscle pairs have a peak in coherence between approximately 130 to 230 degrees. On the other hand, in the elbow-down conditions, only coherence between the anterior and middle deltoids has a distinguishable peak, which occurs around 90 degrees. Interestingly, this peak is absent in the elbow-up conditions.

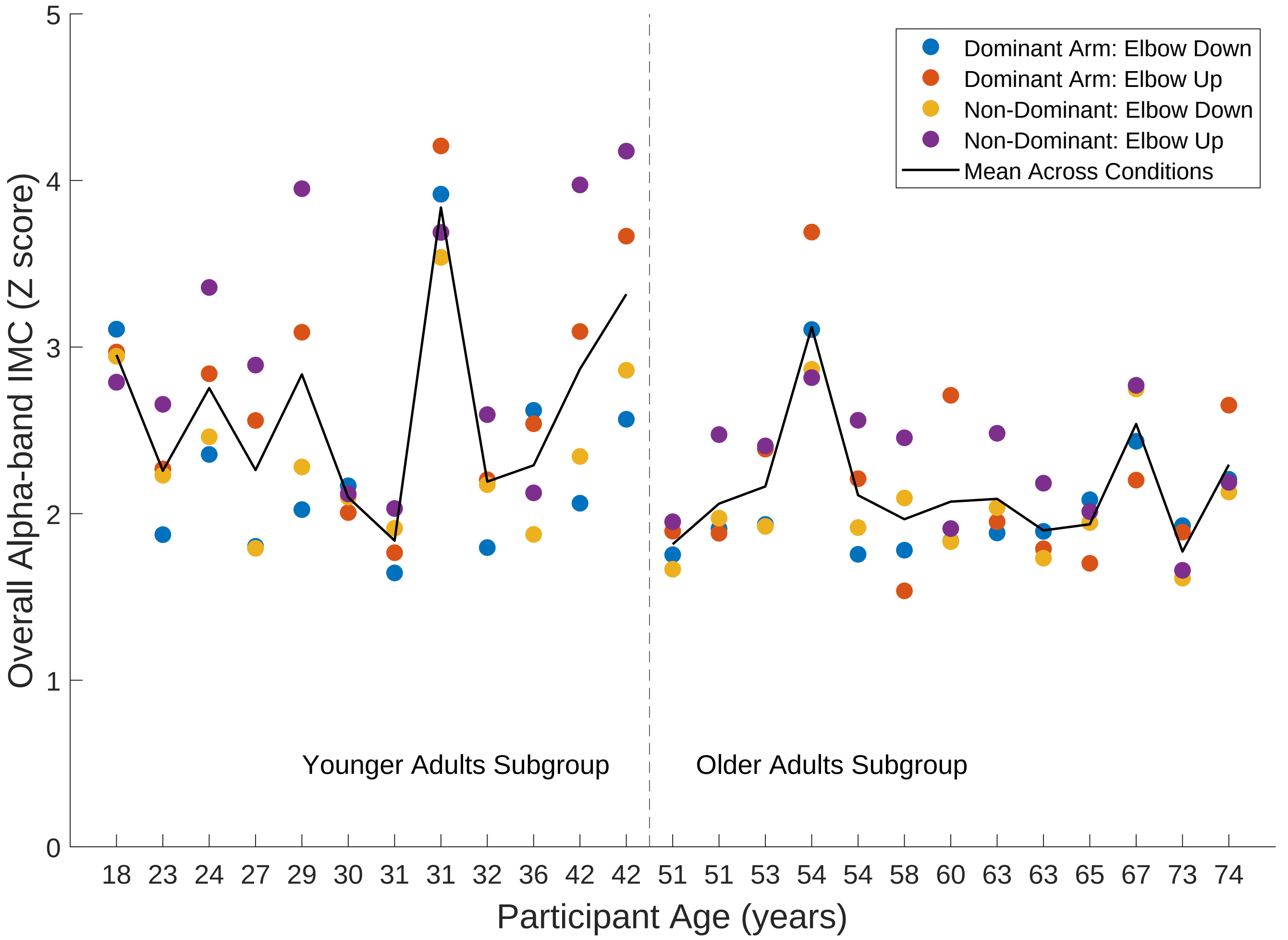

Figure S4. Alpha-band intermuscular coherence averaged across the rotation cycle and muscle pairs (i.e., overall IMC) for each participant and experimental condition. The vertical dashed line delineates participants into younger and older adults as used in age group analyses.

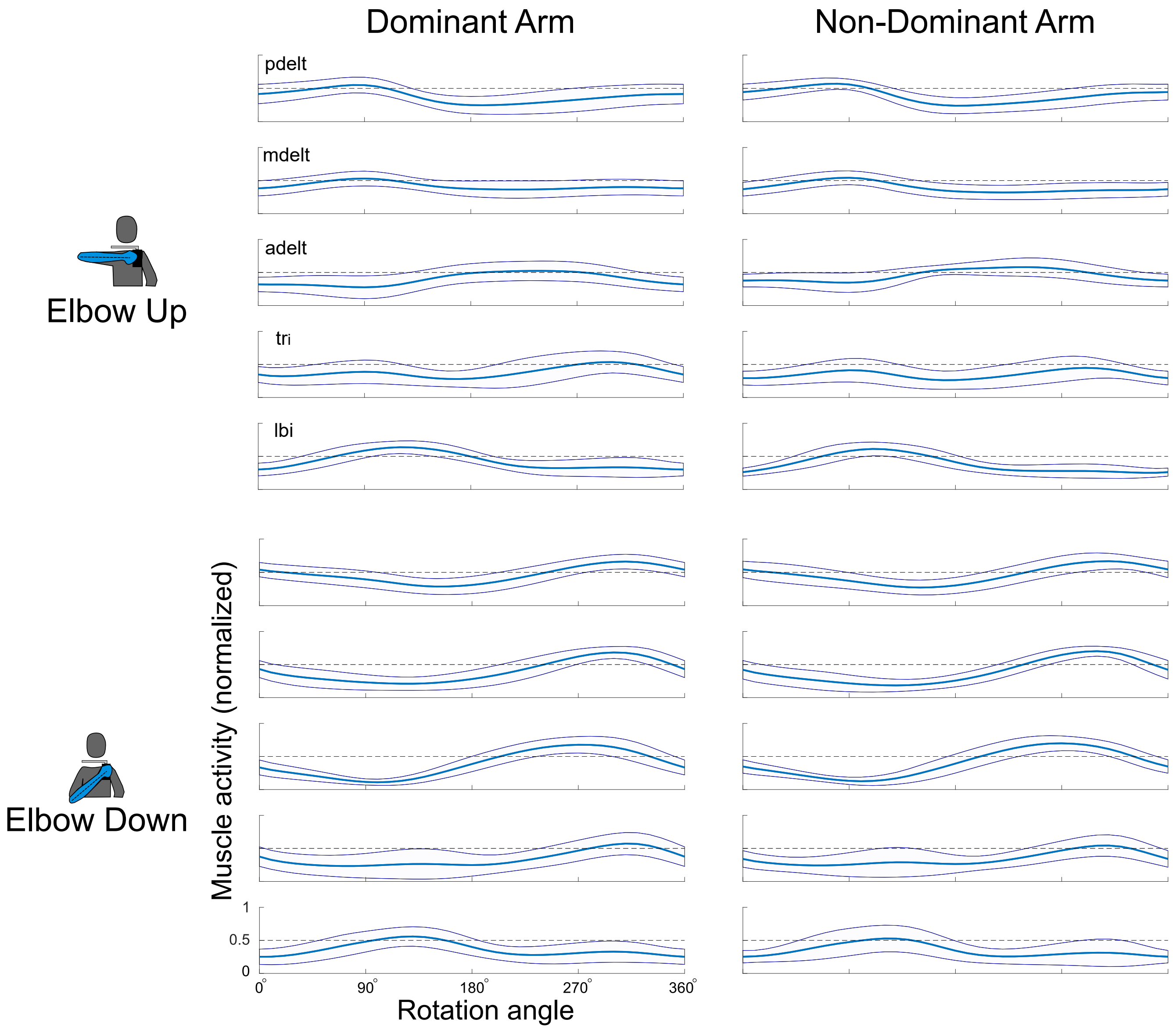

Figure S5. Normalized muscle activity (i.e., EMG-amplitude) binned (10 degree width) across the rotation cycle for all 4 experimental conditions: rotating the ergometer with the dominant (left column) and non-dominant (right column) arms with either the elbow-up (top row) or elbow-down (bottom row). Muscle activity was derived from EMG signals as follows: 1) processed by a digital 4th-order high-pass Butterworth filter with a 250 Hz cutoff frequency and zero-phase lag, 2) rectified by the absolute value function, 3) normalized to unit magnitude, 4) convolution with a 500 ms length Gaussian window, and then 5) binned by taking the average of time-series data concatenated into 10 degree-width bins based on ergometer rotation angles. Each sub figure plots the grand mean and standard deviation across participants binned EMG-amplitude data. The grand mean is depicted by the thickest solid line in the middle of two thinner outer lines with shading that represent a single standard deviation from the grand mean. Dashed lines are provided at a muscle activity amplitude of 0.5 for reference.

2 SUPPLEMENTARY RESULTS: SIGNIFICANT DIFFERENCE BETWEEN INDIVIDUAL MUSCLE PAIRS

2.1 Alpha-band IMC

Contrasts between the elbow-up and elbow-down shoulder postures with the *dominant arm* revealed significant differences for alpha-band IMC between the long head of the biceps and the middle deltoid (0.69 ± 0.38), long head of the biceps and posterior deltoid (0.49 ± 0.28), lateral head of the triceps and middle deltoid (0.63 ± 0.35), lateral head of the triceps and poster deltoid (0.85 ± 0.31), anterior and middle deltoid (-0.93 ± 0.33), as well as between the middle and posterior deltoid (0.78 ± 0.45) muscles. For the *non-dominant arm*, the same contrast of shoulder posture revealed significant differences between the long head of the biceps and anterior deltoid (0.35 ± 0.22), long head of the biceps and middle deltoid (0.86 ± 0.31), long head of the biceps and posterior deltoid (0.39 ± 0.18), lateral head of the triceps and middle deltoid (1.09 ± 0.51), lateral head of the triceps and posterior deltoid (1.09 ± 0.38), anterior and middle deltoid (-0.73 ± 0.33), as well as between the middle and posterior deltoid (0.93 ± 0.36) muscles. Values included in parentheses related to these contrasts report grand mean effect sizes plus or minus the half-width of the 95% two-tail confidence interval. On the other hand, both **contrasts between the dominant and non-dominant arms for the same shoulder posture** had at most small (< 0.5).

2.2 Beta-band and Gamma-band IMC

Five muscle pairs with significant differences in grand mean IMC (averaged across the rotation cycle) were the same across all three frequency bands; namely, lbi with mdelt, lbi with pdelt, tri with mdelt, tri with pdelt, and mdelt with pdelt. Significant differences in IMC were found between lbi with tri, but only in the beta and gamma-bands. Further, significant differences in adelt with mdelt were present in the alpha-band for both arms, but only the dominant arm in the beta-band; while significant differences between adelt and lbi (all bands) as well as adelt and tri (beta and gamma-bands) were present only in conditions with the non-dominant arm. For muscle pairs with significant differences, the postural contrast of IMC for tri with mdelt as well as lbi with mdelt had at least a medium effect size (> 0.5) in all frequency bands; while tri with lbi had at least a medium effect size but only in the beta- and gamma-bands.
